# O036: Antibiotic resistance and molecular epidemiology of panton valentine leukocidin positive methicillin-resistant staphylococcus aureus (PVL+-MRSA): an international survey

**DOI:** 10.1186/2047-2994-2-S1-O36

**Published:** 2013-06-20

**Authors:** M Macedo-Vinas, J Conly, P Francois, R Aschbacher, D Blanc, G Coombs, G Daikos, B Dhawan, J Empel, J Etienne, A Figueiredo, G Golding, L Han, L Hoang, H Kim, R Köck, A Larsen, F Layer, J Lo, T Maeda, M Mulvey, A Pantosti, T Saga, J Schrenzel, A Simor, R Skov, M Van Rijen, H Wang, Z Zakaria, S Harbarth

**Affiliations:** 1 PVL+-MRSA Global Survey Study Group, Geneva, Switzerland

## Introduction

PVL is usually associated with non multiresistant community-acquired MRSA. However, the epidemiology of PVL+-MRSA is changing.

## Objectives

We describe the resistance patterns and molecular epidemiology of PVL+-MRSA isolates from 17 countries around the world.

## Methods

Retrospective laboratory-based survey from 2008 to 2010. Participating countries (Australia, Brazil, Canada, China, Denmark, France, Germany, Greece, Hong Kong, India, Italy, Japan, Korea, Malaysia, Netherland, Poland, Switzerland) reported on susceptibility to 10 non-beta-lactam antibiotics and MLST and/or *spa* typing of PVL+ MRSA isolates. A selection of 49 isolates were analysed by MLVA.

## Results

Overall, susceptibility data of 3236 was reported. The lowest susceptibility was observed for erythromycin in all regions (17.3% in the Americas and 60% both in Europe and Asia&Oceania). Vancomycin-intermediate isolates were reported from Hong Kong and The Netherlands. Multiresistance (3 or more non-beta-lactams) was reported from all regions. Isolates belonged to 8 clonal complexes. ST30 was reported worldwide, being the most frequent type in Brazil and Asia. ST8 was the most frequent in Canada. In Europe, ST80 and ST8 were both reported in the first place. Six major clusters were discriminated by this method, showing a certain geographic specificity and agreement with MLST.

## Conclusion

PVL+-MRSA remains frequently susceptible to non-beta-lactam agents, with in vitro activity of vancomycin, rifampicin, cotrimoxazole and linezolid. However, multiresistant isolates were reported from all regions. Our results are in agreement with recent observations that suggest that USA300 and related clones are gradually replacing the European ST80 clone. It is imperative to continue monitoring susceptibility patterns and molecular epidemiology of MRSA to provide clinicians with the most up-to-date information.

## Disclosure of interest

M. Macedo-Vinas: None declared, J. Conly Grant/Research support from Alberta Heritage Foundation for Medical Research, the Canadian Institutes for Health Research and Pfizer, Speaker's Bureau of Janssen-Ortho and Pfizer, Consultant for Canadian Agency for Drugs and Technologies in Health , P. Francois: None declared, R. Aschbacher: None declared, D. Blanc: None declared, G. Coombs: None declared, G. Daikos: None declared, B. Dhawan: None declared, J. Empel: None declared, J. Etienne: None declared, A. Figueiredo: None declared, G. Golding: None declared, L. Han: None declared, L. Hoang: None declared, H. Kim: None declared, R. Köck: None declared, A. Larsen: None declared, F. Layer: None declared, J. Lo: None declared, T. Maeda: None declared, M. Mulvey: None declared, A. Pantosti: None declared, T. Saga: None declared, J. Schrenzel Consultant for bioMérieux, A. Simor Grant/Research support from Pfizer Canada, Speaker's Bureau of Pfizer Canada, Novartis, and Sunovion, R. Skov: None declared, M. Van Rijen: None declared, H. Wang: None declared, Z. Zakaria: None declared, S. Harbarth Grant/Research support from B. Braun, Pfizer and the European Commission (MOSAR network contract LSHP-CT-2007-037941), Speaker's Bureau of bioMérieux and Pfizer, Consultant for Destiny Pharma, DaVolterra and bioMérieux

